# Estimating Prion Adsorption Capacity of Soil by BioAssay of Subtracted Infectivity from Complex Solutions (BASICS)

**DOI:** 10.1371/journal.pone.0058630

**Published:** 2013-03-04

**Authors:** A. Christy Wyckoff, Krista L. Lockwood, Crystal Meyerett-Reid, Brady A. Michel, Heather Bender, Kurt C. VerCauteren, Mark D. Zabel

**Affiliations:** 1 Department of Microbiology, Immunology and Pathology, College of Veterinary Medicine and Biomedical Sciences, Colorado State University Prion Research Center, Fort Collins, Colorado, United States of America; 2 National Wildlife Research Center, Wildlife Services, United States Department of Agriculture, Fort Collins, Colorado, United States of America; University of Utah School of Medicine, United States of America

## Abstract

Prions, the infectious agent of scrapie, chronic wasting disease and other transmissible spongiform encephalopathies, are misfolded proteins that are highly stable and resistant to degradation. Prions are known to associate with clay and other soil components, enhancing their persistence and surprisingly, transmissibility. Currently, few detection and quantification methods exist for prions in soil, hindering an understanding of prion persistence and infectivity in the environment. Variability in apparent infectious titers of prions when bound to soil has complicated attempts to quantify the binding capacity of soil for prion infectivity. Here, we quantify the prion adsorption capacity of whole, sandy loam soil (SLS) typically found in CWD endemic areas in Colorado; and purified montmorillonite clay (Mte), previously shown to bind prions, by BioAssay of Subtracted Infectivity in Complex Solutions (BASICS). We incubated prion positive 10% brain homogenate from terminally sick mice infected with the Rocky Mountain Lab strain of mouse-adapted prions (RML) with 10% SLS or Mte. After 24 hours samples were centrifuged five minutes at 200×g and soil-free supernatant was intracerebrally inoculated into prion susceptible indicator mice. We used the number of days post inoculation to clinical disease to calculate the infectious titer remaining in the supernatant, which we subtracted from the starting titer to determine the infectious prion binding capacity of SLS and Mte. BASICS indicated SLS bound and removed ≥ 95% of infectivity. Mte bound and removed lethal doses (99.98%) of prions from inocula, effectively preventing disease in the mice. Our data reveal significant prion-binding capacity of soil and the utility of BASICS to estimate prion loads and investigate persistence and decomposition in the environment. Additionally, since Mte successfully rescued the mice from prion disease, Mte might be used for remediation and decontamination protocols.

## Introduction

Prions are infectious agents of transmissible spongiform encephalopathies (TSEs) [1]. Misfolded, pathologic isoforms (PrP^Sc^) of the normal mammalian prion protein (PrP^c^) associate with prion infectivity, generally resist protease degradation, and often form insoluble, amyloidogenic aggregates [2]. Prions are capable of horizontal transmission between animals and indirect transmission from contaminated environments [3]–[10]. For reasons that remain unclear, indirect environmental transmission of prions appears to be limited to scrapie and chronic wasting disease (CWD) prions, and does not appear to be an ecological component of bovine spongiform encephalopathy (BSE) or other TSEs. This phenomenon may relate to scrapie and CWD sharing similar lymphotropic, shedding and transmission characteristics [11]–[13]. Infectious prions are likely deposited into the environment through alimentary shedding [14], [15], placental material [16], antler velvet deposits [17] and the decomposition of prion-positive mortalities [5]. Once in the environment, studies have shown PrP^Sc^ to adsorb strongly to soil components [18]–[20], remain infectious [21]–[23] and persist for years [5], [7], [21], [24]. Indirect transmission most likely occurs through incidental and geophagic ingestion of soil or other contaminated fomites, as well as deer sign-post behavior such as scraping and marking overhanging branches [5], [8], [25].

Experimental evidence suggests that the particularly strong adsorption relationship of prions to soil colloids, or clays (defined as particles <4 µm), may be responsible for the longevity in the environment [18], [19]. Studies have shown percent-clay content of soil significantly influences the cation exchange capacity of soil and its overall negative charge [26]. Electrostatic and hydrophobic interactions between the prion protein and clay are thought to mediate this non-specific adsorption activity [27]–[30]. Specifically, montmorillonite (Mte), the most commonly occurring smectite clay, has been most implicated in the adsorption of prions in the environment [31]. Mte is a 2∶1 phyllosilicate clay consisting of 2 tetrahedral silica composed molecules flanking one octahedral aluminum composed molecule, forming a sheet. An interlayer space exists between sheets capable of expanding to >2 nm depending on the cationic concentration of the solution. It has been hypothesized that prions may enter this interlayer area like other proteins. However, Johnson et al. [31] did not find evidence of this in their experimental system and other studies suggest extensive protein unfolding would be required [32], which is unlikely for PrP^Sc^. Mte is prevalent throughout the US mountain west, including CWD endemic areas [33], [34]. Models suggest that the prevalence of Mte at a landscape level may explain and predict CWD prevalence, which can exceed 20% in free-ranging cervids [33], [35].

Other soil components such as organic material, quartz, tannins and humic acid have also been implicated in prion adsorption [19], [29], [31], [36]–[39]. Whole soil also includes highly reactive humic substances, which have large specific surface areas and high binding capacities [40]. Humic acid can coat mineral surfaces imparting a net negative charge [41]. However, due to the unknown tertiary structure of PrP^Sc^, specific interactions and adsorption dynamics to soil and humic substrates have yet to be identified.

The robust adsorption relationship between the prion protein and soil has proven difficult to measure or reverse, limiting prion detection sensitivity, estimation of prion adsorption capacity of soil [19], [29], [31], [42] and general progress in studying prions in the environment. Additionally, prion detection in soil has been successful only in laboratory experiments using a variety of different methods including antibody labeling [38], electrophoresis [37], bioassay [22], detergent extraction [36] and protein misfolding cyclic amplification (PMCA) [22], [43].

To date, hypothesized soil interactions have largely been demonstrated with recombinant prion proteins [27], [29], [36], which probably interact differently with soil than glycosolated misfolded, aggregated prions. Previous investigations of the soil-prion relationship using whole brain homogenates containing mouse and hamster adapted prions have attempted to quantify the amount of PrP^Sc^ bound to soil [19], [31], [44]. But PrP^Sc^ does not necessarily correlate with prion infectivity and studies estimating infectivity using prion-bound soil fractions have produced conflicting data. Soil-bound prions apparently increase infectivity upon oral inoculation [22], but decrease infectivity upon intracerebral (i.c.) inoculation [23]. To circumvent these issues, and more accurately quantify infectious prion binding capacity of soil, we developed a converse assay. We investigated the adsorption capacity of prions to soil using an infectivity subtraction assay of titrated prion strains. This methodology allows measurement of unbound and unadulterated prions instead of prions bound to soil, which can alter infectivity [22], [23]. We calculated the adsorption capacity of two soil types, a whole Colorado sandy loam soil (SLS) and pure montmorillonite clay (Mte) by assaying residual infectivity of supernatants from prion-soil matrices using TgA20 mouse bioassay [45]. SLS bound over 95% of prion infectivity and Mte bound over 99.99% prion infectivity. These data promote BASICS as an effective tool for quantifying prion adsorption to soil as a function of infectivity and Mte as a potential compound for bioremediation of prion-contaminated solutions. We further propose that BASICS can improve estimates of landscape contamination that might exist in scrapie or CWD endemic areas, thereby enhancing our understanding of the larger issues of environmental prion persistence.

## Methods

### Mice

TgA20 mice over-expressing mouse PrP^c^ were generated as previously described [45] and allowed for quantitative LD_50_ infectivity analysis [46]–[48], defined as the prion dose that kills half of inoculated mice.

### Ethics Statement

Mice were bred and maintained at Lab Animal Resources, accredited by the Association for Assessment and Accreditation of Lab Animal Care International, in accordance with protocols approved by the Institutional Animal Care and Use Committee at Colorado State University (Protocol ID: 09-1580A). Intracerebral inoculations were performed under Isoflurane anesthesia, and mice euthanized using CO_2_ inhalation to effect followed by decapitation. All efforts were made to minimize suffering.

### Soil

Whole SLS used for this study was sourced from a private ranch in Southern Colorado located on the eastern side of the Rocky Mountains and within a game management unit which continues to test negative for CWD in free-ranging cervid populations [49]. Soil was collected with the land owner's (A. C. Wyckoff) permission, no additional permissions or permits were required for the described field studies. SLS was passively air-dried, serially sifted first through a 1 cm sieve to remove rocks and debris, then through a 2 mm sieve and autoclaved (dry soil, 90 min at 120°C) to reduce incidental biotic agents naturally present in soil. Montmorillonite (powdered Western Bentonite) was sourced from Panther Creek, Co and supplied by Ward’s Natural Science (San Luis Obispo, Ca).

### Soil Analyses

Soil classification analysis of whole soil was conducted by the Colorado State University Soil, Water and Plant Testing Laboratory (Fort Collins, Co). X-ray diffraction mineralogy analysis of whole soil was conducted by K-T GeoServices, Inc. (Gunnison, Co). Whole soil analysis included XRD weight percentage for bulk (whole rock) and clay fraction (<4 µm), pH, percent organic material, and soil texture classification of basic elements (Table 1). The following definitions were used for clay mineral classification: *Mixed-Layer Illite/Smectite* – A clay mineral group containing interlayered or interstratified Illite and Smectite. Mixed layer type was identified by the minerals involved (Illite and Smectite), the type of order or stacking along the Z-axis (random or not ordered), and the proportions of the minerals involved (10% Illite and 90% Smectite). *Illite and Mica* – Common non-expanding minerals which are hydrated silicates containing potassium, silica and aluminum. *Kaolinite* and *Chlorite* – Common non-expanding hydrous aluminum silicate clay minerals. Montmorillonite clay was not further analyzed, specifics were obtained from the Material Safety Data Sheet (MSDS) sheet provided by the supplier.

**Table 1:** Soil Component Analysis.

*Mineral*	*Whole Soil* a	*Mte* a
Quartz	35.9	
K-Feldspar	9.3	
Plagioclase	38.3	
Amphibole	1.3	
Calcite	1.3	
Pyrite	1.6	
Hematite	0.8	
R0 M-L I/S 90S^b^	2.2 (19.1)^c^	100.0
Illite & Mica^b^	7.7 (67.0)	
Kaolinite^b^	1.4 (12.2)	
Chlorite^b^	0.2 (1.7)	
***Total***	100.0 (11.5)^d^	100.0
***Soil Characteristics***		
Texture class	Sandy Loam	clay
% Sand	72.0	NA^f^
% Silt	14.0	NA
% Clay	14.0	100.0
Ph	7.5	9.9
EC (mmhos/cm)^e^	4.6	NA
% Organic Material	3.6	0

a % weight of whole SLS.

b clay classification.

c % of total clay weight.

d clay weight % of total.

e electrical conductivity (EC), measurement of salinity.

f NA, not applicable.

### Sources and Preparation of Prion Inocula

The Rocky Mountain Lab passage 5 strain of mouse-adapted scrapie (RML5) was previously described [50]. We derived the TgA20RML strain by passaging RML5 into TgA20 mice, resulting in inoculum with approximately one log lower infectivity titer compared to the original RML5 (see Table 2). Brain homogenates of clinically ill mice were prepared to 10% dilution in PMCA buffer (4 mM EDTA, 150 nM NaCl in PBS) and further diluted to 1% into similarly prepared 10% TgA20 normal brain homogenate (NBH) as previously described [51].

**Table 2:** Incidence and infectivity titers of prion inocula before and after adsorption.

Inoculum	Adsorbed to	Incidence^a^(mean ± SD DPI^b^)	Infectivity Titer^c^			% bound
			Input^d^	unbound	bound	
NBH	SLS	0/2 (non-clinical at 250)	0	0	0	0
TgA20RML	nothing	4/4 (82±3.4)	1.70	NA^e^	NA	NA
	SLS	6/6 (99±6.4)	1.70	0.06	1.64	96.45
	Mte	0/7 (non-clinical at 200)	1.70	0	1.70	100
RML5	nothing	4/4 (73±13.5)	14.8	NA	NA	NA
	SLS	7/7 (87±6.6)	14.8	0.68	14.1	95.32
	Mte	1/5 (109, 4 mice non-clinical at 200)	14.8	<0.0032^f^	≥14.8	99.98

a number of terminally ill mice/number infected.

b DPI, days post infection.

c ×10^4^ mean LD_50_ after 24 h @ 23°C. All SDs ≤0.001×10^4.^

d Initial titer of inocula prior to adsorption.

e NA, not applicable.

f below linear range of bioassay.

### BioAssay of Subtracted Infectivity from Complex Solutions (BASICS)

**Figure 1:**
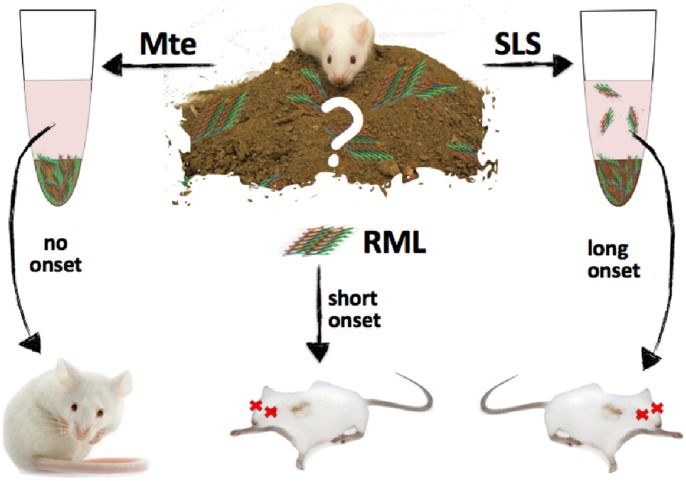
Visual Schematic of BASICS. To determine prion binding capacities of Mte and SLS, we incubated known titers of RML prions with or without Mte or SLS for 24 h at 23°C. A brief, low-speed centrifugation separated bound prions in the pellet from unbound prions in the supernatant. Degree of prion binding is then measured by bioassay in susceptible mice, subtracting supernatant titers from Mte (virtually no disease onset) or SLS (long onset) bound samples from control RML (short onset) supernatant titers.

We performed an infectivity subtraction assay to estimate binding capacity of SLS and Mte soil (Figure 1). We prepared 10% w/vol soil solutions by adding dry soil to previously prepared 10% brain homogenates (e.g. 30 mg dry soil added to 270 µl homogenate). All inocula, with and without soil, were incubated at 23°C for 24 hours on a rocker to balance maximal binding in a competitive matrix with the decomposition of brain homogenate [19], [28], [31]. Samples were then centrifuged for 5 min at 200×g (Accuspin Micro, Fisher Scientific, Waltham, Ma) to clarify solutions of soil particles, thereby subtracting prion infectivity bound to soil or Mte from prion infectivity remaining in supernatant. Inoculation groups included non-soil treated TgA20RML and RML5 to establish baseline infectivity titers, experimental treatments of TgA20RML and RML5 with SLS or Mte soil, and a negative control of NBH with SLS.

Anesthetized mice were intracerebrally inoculated with 30 µl of inoculum (with 1% Pen-Strep added) as previously described [51]. Each treatment groups consisted of 5–7 mice. Onset of clinical disease was measured by scoring mice from normal (0) to exhibiting terminal clinical signs (4) for 7 different clinical signs including ataxia, akinesia, hyperactivity (0–3 scale), extensor reflex, tail rigidity (0–2 scale), weight loss and tremors. Mice receiving a composite score of 9 or greater, a single clinical score of 4, or exhibiting paralysis were euthanized and days post inoculation (DPI) to clinical disease recorded. DPI was used to calculate log infectivity titers of each inocula based on previous LD_50_ determinations for RML in TgA20 mice [2], [45], [52]. We used the linear equation *y* = 11.45–0.088*x* (*y*, logLD_50_ per gram of brain; x, incubation time in DPI to terminal disease) to calculate infectivity titers as outlined in Reed and Muench [48]. Several non-clinical mice from each Mte-inoculated group were also euthanized after 130 DPI, and 200 DPI (the end of the study) to test their brain tissue for sub-clinical levels of prions by serial protein misfolding cyclic amplification (sPMCA). Brains tissues were collected from all mice for western blot (stored at −80°C) and a subset of mouse brains were also sampled for histological analysis (2/3 of brain was fixed in 10% formaldehyde, remaining section was frozen). Statistical analysis of Kaplan-Meyer survival curves and Student’s t-tests of log infectivity were conducted using Prism 5 (GraphPad, La Jolla, Ca).

### sPMCA and Western Blotting

Brain tissues of clinical and non-clinical mice, as well as samples of each inoculum were tested by western blotting before and after sPMCA. Prior to assay, brain tissues collected from mice were homogenized following the methods of Meyerett et al. [51]. sPMCA amplification substrate consisted of 25 µl of 10% TgA20 NBH combined with 25 µl of sample in 0.2 ml tubes. Tubes were sealed with parafilm, loaded into a holding tray and placed in a 37°C water bath in the Misonix 4000 sonicator horn (Qsonica Inc., Farmingdale, NY). Samples were sonicated at approximately 200 watts (70% max power) for 40 sec every 30 min for 24 h, constituting one round. For each subsequent round, 25 µl of each sample from the previous round was added to 25 µl of fresh NBH. Duplicates of each sample were subjected to 6 rounds of PMCA to balance desired sensitivity (>80% of 10^−7^ dilution prion samples detected positive) and specificity (>98% of NBH samples remain negative) of the detection assay. Each group of samples was processed with at least five NBH negative controls and one positive plate control (CWD positive elk brain homogenate E2, 1∶10,000).

For visualization by western blot, 18 µl of sample was digested with 2 µl of 50 µg/ml proteinase K (PK, Roche, Basel, Switzerland) for 30 min at 45°C. The reaction was stopped by adding lithium dodecyl sulfate sample loading buffer (Invitrogen, Carlsbad, Ca) and boiling samples for 5 min at 95°C. Samples were electrophoresed through 12% sodium dodecyl sulfate polyacrylamide gels (Invitrogen) then electro-transferred to Immobilon P^SQ^ PVDF membranes (Millipore, Billerica, Ma) in transfer buffer (Invitrogen). Membranes were blocked for 1 hr with 5% nonfat milk in PBS with 0.1% Tween 20, and incubated overnight at 4°C in Superblock (Pierce, Waltham, Ma) with HRP-conjugated anti-PrP Bar-224 monoclonal antibody (SPI bio) diluted 1∶20,000. Blots were washed 6×10 min in PBS with 0.2% Tween 20 before visualizing proteins using Immobilon chemiluminescent substrate (Millipore) and a Fujifilm LAS 3000 gel documentation system.

### Immunohistochemistry

Dissected tissues were prepared and stained for PrP^Sc^ detection as previously described [51] with the following modifications. Briefly, tissues were treated with DAKO target retrieval solutions (DAKO, Carpinteria, Ca), then with formic acid to degrade PrP^c^. PrP^Sc^ was labeled with anti-PrP BAR224 followed by incubation with secondary EnVision HRP-conjugated anti-mouse antibody that was visualized with chromagen 3-Amino-9-ethylcarbazole (DAKO). Hemotoxylin and Glial fibrillary acidic protein (GFAP) stain of activated astrocytes was performed by the Colorado State University Histology and Diagnostic Laboratory as previously described [51]. Briefly, slides were treated with DAKO target retrieval solution then treated with primary anti-GFAP rabbit antibody at 1∶100 (Cell Marque, Rocklin, Ca). Secondary anti-rabbit-goat biotinillated antibody was used with (BioGenex, San Romano, Ca) Enhanced Alkaline Phosphatase Red Detection Kit (Ventana, Tucson, Az).

## Results

In this study we collected soil from an area in southern Colorado with similar soil composition to CWD endemic areas but with no reported cases of CWD. Soil component analysis revealed clay content similar to that found in areas of Colorado exhibiting high prevalence (Table 1). Specifically, the smectite clay Mte, previously shown to avidly bind prions [31] constituted approximately 2% of total soil and 19.1% of total clay content in our samples.

**Figure 2:**
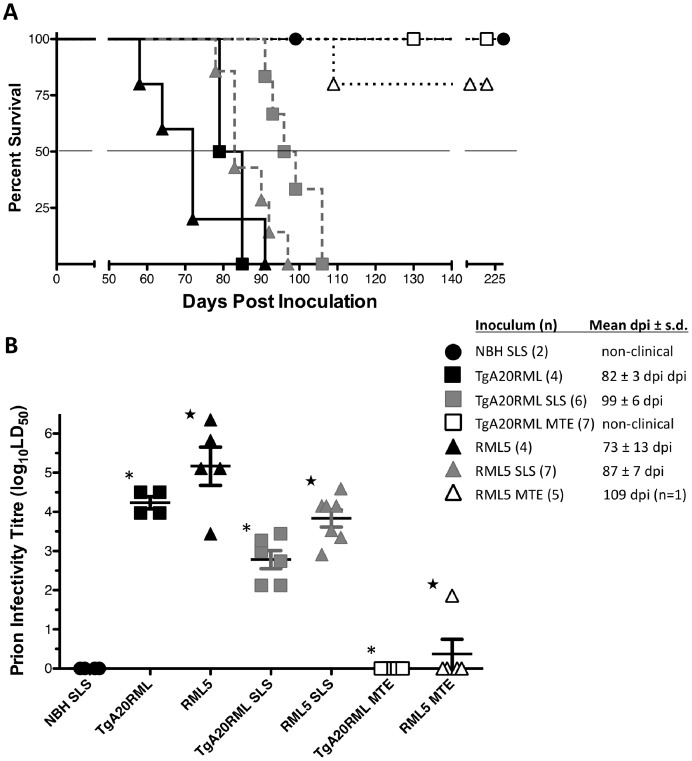
Survival of TgA20 indicator mice following i.c. inoculations. (**A**) Kaplan-Meyer survival curve of 7 treatment groups demonstrates the delayed disease onset in mice infected with SLS treated inocula (grey squares and triangles), and the nearly complete abrogation of disease in mice infected with Mte treated inocula (open squares and triangles) compared to control mice infected with neat inocula (black squares and triangles). Mice infected with SLS-treated negative brain homogenate (black dots) did not exhibit any disease. (**B**) Disease onset of each group were clustered and consistent with reduced LD_50_ values. Inoculum type was significantly different (P<0.05) than their respective treatment, (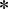 = significant difference between TgA20RML treatments, ★ = significant differences between RML5 treatment groups). Data is presented with treatment group median and s.d. error bars.

Incubation of 1% TgA20RML and 1% RML5 prions with either SLS or Mte significantly reduced the bioassay infectivity, resulting in delayed clinical disease (p<0.05, Figure 2 and Table 2). Specifically, SLS incubation reduced the bioassay infectivity of the TgA20RML by 28.2 fold, a 96.5% reduction in infectivity. Infectivity of the same inoculum incubated with Mte was below bioassay detection limits (130 DPI [46]), resulting in all mice surviving to the end of the study at 200 DPI with no clinical signs of disease. Likewise, the infectivity of the RML5 inoculum was reduced by 21.4 fold, or 95.3%, after incubation with SLS. The mean binding capacity of SLS for RML prions in both inocula was 8.13×10^5^±1.2 LD_50_ units/g soil. Incubation of RML5 with Mte resulted in a near total removal of infectivity with only one mouse becoming ill, equating to at least a 1380-fold reduction in infectivity. Mte completely removed lethal doses of TgA20RML prions (1.7×10^4^ LD_50_ units), indicating that its RML5 binding capacity is at least 5.63×10^8^ LD_50_ units/g of Mte.

**Figure 3:**
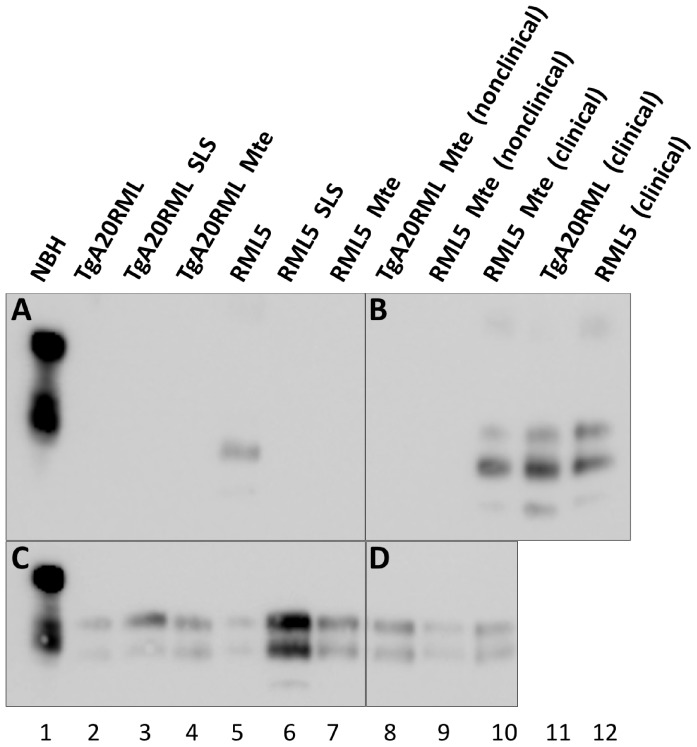
Representative PK digestion and Western blot analyses of inocula and inoculated animals. All samples were PK digested except lane 1 (**A**) PrP^Sc^ content of inocula were below western blot detection levels with the exception of RML5 (lane 5). (**B**) Brain homogenates from non-clinical experimental animals (lanes 8–9) were also negative by western blot, however, samples from clinically ill mice showed PrP^Sc^ (lanes 10–12). (**C**) With 6 rounds of PMCA, PrP^Sc^ was detected in all inocula, and (**D**) in non-clinical mouse brain tissues samples.

**Figure 4:**
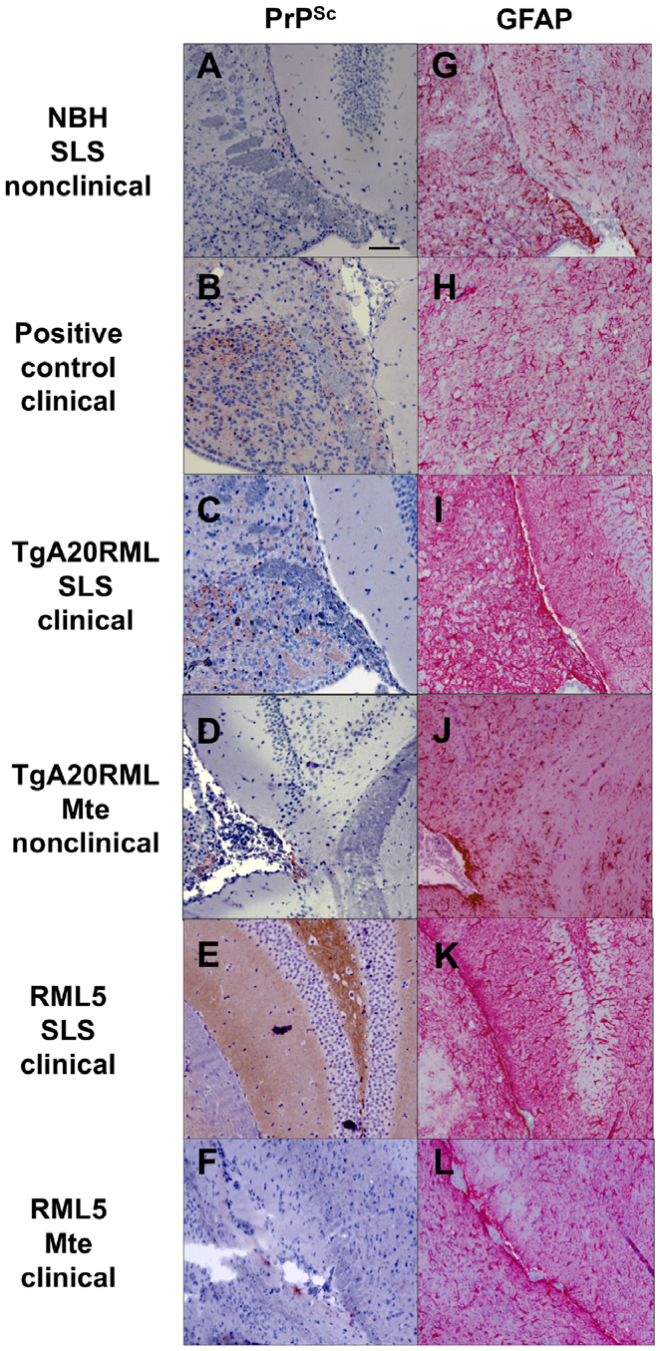
Representative Histology of TgA20 indicator mice. Selected examples of histological analysis using immunohistochemistry with PrP specific BAR224 Ab (reddish-brown staining, panels A–F) and anti-GFAP antibody staining activated astrocytes (bright red, panels G–L) in hippocampal sections. (**A and G**) Negative control sections from mice inoculated with SLS-treated NBH exhibited no PrP^Sc^ staining or spongiosis and limited astrocyte activation. (**B and H**) Positive control sections from mice inoculated with RML5 revealed diffuse PrP^Sc^ staining and significant spongiosis and astrogliosis. (**C and I**) Sections from mice inoculated with SLS-treated TgA20RML resulted in limited PrP^Sc^ deposits, spongiosis and astrogliosis, while (**D and J**) sections from mice inoculated with Mte-treated TgA20RML revealed little or no scrapie neuropathology. (**E and K**) sections from mice inoculated with SLS-treated RML5 resulted in neuropathology similar to sections from TgA20RML treated mice (Band H). (**F and L**) Hippocampal sections from the only mouse to become ill with Mte treated RML5 showed limited PrP^Sc^ spongiosis and astrogliosis.

To determine whether non-clinically sick mice replicated sub-clinical levels of prions, we attempted to amplify minute quantities of prions from their brains using sPMCA. We detected prions in 2/7 brains from non-clinical mice inoculated with TgA20RML pre-adsorbed with Mte and 2/4 brains from non-clinical mice inoculated with Mte-adsorbed RML (Figure 3, Table 3). These results suggested that sub-clinical levels of prions existed in some individuals despite the lack of clinical disease. To confirm this observation, we also investigated neuropathology in these mice and compared them to clinically ill mice. Histological examination for PrP^Sc^ deposits, spongiosis and astrogliosis revealed differences in histopathology between SLS-adsorbed prion-inoculated mice and the single Mte-adsorbed RML5-inoculated mouse that became clinically ill (Figure 4). We detected no PrP^Sc^ or spongiform lesions and only mild astrogliosis in brains from non-clinical mice inoculated with control NBH (panels A and G) and Mte-adsorbed TgA20RML (D and J). We detected small deposits of PrP^Sc^ and spongiosis and slightly more astrogliosis in the brain of the lone clinically sick mouse inoculated with Mte-adsorbed RML5 (F and L). In contrast, we observed both diffuse and punctate PrP^Sc^ aggregates and mild to severe spongiosis and astrogliosis in brains from clinically ill mice inoculated with non-adsorbed prions (B and H) and whole soil-adsorbed TgA20RML (C and I) and RML (E and K). Together with the biochemical analysis, these data confirm prion infection in clinically ill mice, as well as sub-lethal infection in non-clinical mice, which we now call sub-clinically ill mice.

**Table 3:** Disease status and detection of prions in non-clinical mice inoculated with Mte-treated-inocula.

Treatment Mice	DPI	sPMCA +/−
TgA20RML-Mte		
1	131	**+**
2	131	**−**
3	131	**−**
4	200	**+**
5	200	**−**
6	200	**−**
7	200	**−**
RML5-Mte		
1 (clinical)	109	**+**
2	131	**−**
3	131	**−**
4	200	**+**
5	200	**+**

## Discussion

Environmental persistence and increased transmissibility of soil bound prions remain poorly understood but extremely important aspects of both scrapie and CWD ecology. The use of SLS and Mte allowed us to model the complexity of prion binding in the natural environment, while estimating the relative contribution of a soil component previously shown to avidly bind prions. The use of RML in these studies allowed for LD_50_ calculations and quantitative statements of prion binding capacity of soil and Mte as a function of infectivity. Use of whole brain homogenate, as opposed to recombinant protein or enriched prions, accounts for the complexity of the tissue and competitive matrix binding activity [19]. PrP^Sc^ is conceivably a small component of the brain matrix, and will compete with other proteins for binding sites on soil particles. Previous studies revealed the potential for increased adsorption if allowed to incubate for more than 24 hours [19], so we consider the adsorption measurements in our study to be an conservative estimate of the adsorption capacity of soil in a natural system. However, as previously mentioned, we sought to balance decomposition and microbial contamination of tissues with binding activity. Surprisingly, we observed a nearly one log reduction in infectivity of the positive control, non-adsorbed, inocula simply from an 24-hour incubation at room temperature. We also acknowledge that the behavior of RML in soil may not fully represent the behavior of scrapie or CWD in soil [44], [53]. We used RML as a model system because RML titers have been previously determined. Other recent prion-soil binding studies used titered hamster scrapie strains to estimate [31] and quantify [23] prion binding capacity of soil. However, both studies involved inoculating soil bound prions, which exhibited different infectivity than equivalent doses of unbound prions and varied by inoculation route [23]. These factors potentially skewed estimates of infectious doses adsorbed to soil.

BASICS circumvents these problems by quantifying prion binding to soil by subtracting residual prion titers present after soil adsorption from initial prion titers before soil adsorption. We are currently titrating several other CWD field isolates and laboratory strains and will use BASICS to quantify binding capacity of relevant soil types to relevant prion strains.

We found a dramatic decrease in infectivity with a simple 24-hour soil incubation. The Mte treatment of TgA20RML bound sufficient amounts of infectious prions to prevent disease onset entirely. Similar binding was seen in RML5 samples, resulting in SLS binding 95.3% of infectious prions and the Mte binding ≥99.98%. These results suggest that the adsorption capacity of the Mte, in these experimental conditions, lies somewhere between the TgA20RML and RML5 titers. If the Mte comprises the majority of smectite clay in the soil (90% of the 2.2% smectite, Table 1), then we calculate the maximal binding capacity of Mte present in the soil to be 98.51×10^4^ LD_50_ units of RML per gram of soil. This amount comprises between 2% and 18% of the total prion binding capacity of soil that we observed for RML5 and TgA20RML, respectively. The estimated 2% binding capacity of Mte for RML5 correlates to the 2% Mte found in SLS, suggesting that the Mte is saturated with prion infectivity. The observation that the 2% of Mte correlates to binding nine-fold more TgA20RML infectivity (18% of total bound infectivity) in SLS also supports this interpretation, because TgA20RML titers were approximately nine times lower than RML5 titers. This would leave the remaining prions available for binding to other soil components such as other clays, quartz, humic acid or other organic material. Indeed, other soil components have been implicated in protein adsorption, including organic material, tannins, quartz [19], [29], [31], [36]–[39], and competitive matrices have been shown to retard prion binding to soil [19]. Thus, we cannot completely disqualify the effects of small amounts of residual soil components remaining in solution after low-speed centrifugation that may bind prions and decrease their infectivity upon i.c. inoculation. But these effects are likely minor since centrifugation removed clay components, which we show here as others have previously, to be the major prion binders. If such effects exist, we again acknowledge that BASICS would conservatively estimate prion binding capacity of soil.

These data suggest that Mte is not the only factor determining prion binding capacity of soil. However, we propose that prions bind Mte with relatively high avidity and affinity compared to other soil components, whose prion interaction may be more reversible, creating equilibrium between prions bound to soil and free in solution. We hypothesize that Mte concentrations in the soil dictate this equilibrium and likely result in residual infectivity in supernatants in our and other experimental systems [54], [55] and possibly increased mobility in a natural system [56]. Indeed, the neuropathology and sPMCA data revealed sub-clinical levels of prion in the brain tissue of mice inoculated with Mte-adsorbed inoculum. Although the residual prions were not biologically relevant since the mice exhibited no clinical or pathological signs of scrapie, similar subclinical infections in wild cervid populations may contribute to ecologically relevant contamination, persistence and transmission.

Perhaps the most utilitarian finding of this study was the prevention of disease by pre-adsorbing prions with Mte. These data strongly promote Mte for prion remediation applications. Environmental prion mitigation looms as a potential desideratum for agriculture and wildlife management. However, options for degradation and removal of prions have shown limited efficacy [57]–[59]. Our results suggest that the binding of prions to Mte may be utilized for removing prions from liquids. Landscape application might not be feasible, but other decontamination or remediation applications may be possible in the medical, municipal and research sectors. For example, decontamination of blood, urine and components thereof, as well raw water in endemic areas and liquid waste in prion research facilities may be feasible.

In summary, we propose that although constituting a relatively small fraction of total soil, the high binding avidity and affinity of Mte results in high prion occupancy at or near saturation that may drive the likelihood of environmental prion contamination, persistence and transmission in nature, as has been previously suggested [33]. We are currently testing this hypothesis by experimentally increasing Mte concentrations in whole soil and using BASICS to assess the correlation to increased prion binding capacity of soil.
